# What is the impact of sensory practices on the quality of life of long-term care residents? A mixed-methods systematic review protocol

**DOI:** 10.1186/s13643-018-0783-9

**Published:** 2018-08-04

**Authors:** Chantal Backman, Michelle Crick, Danielle Cho-Young, Megan Scharf, Beverley Shea

**Affiliations:** 1 School of Nursing, Faculty of Health Sciences, University of Ottawa, 451, Smyth Road, RGN 3239, Ottawa, ON K1H 8M5 Canada; 2 School of Nursing, Faculty of Health Sciences, University of Ottawa, 451, Smyth Road, Ottawa, ON K1J 8M5 Canada; 3 School of Epidemiology and Public Health, University of Ottawa, 501, Smyth Road, Ottawa, ON K1H 8L6 Canada

**Keywords:** Senses, Quality of life, Long-term care, Systematic review

## Abstract

**Background:**

With age, the acuity of the five senses (i.e., hearing, sight, taste, smell, touch) is reduced. These types of sensory changes can affect day-to-day activities, making it more difficult for individuals to communicate and to interact with the world around them. The five senses allow us to receive information from the environment in the form of sound, light, smell, taste, and touch. As an older person’s senses decline, they need more stimulation to be aware of these sensations. In long-term care settings, appropriate sensory practices are needed to address the diminishing senses of older adults. The objective of this mixed-methods systematic review is to examine the relationship between the sensory practices and the quality of life of residents living in long-term care settings and to develop an aggregated synthesis of mixed-methods studies to derive recommendations for policy, practice, and research.

**Methods:**

We will conduct a mixed-methods systematic review in accordance with the Cochrane Handbook. A search strategy has been developed with an expert health sciences librarian and peer reviewed using Peer Review for Electronic Search Strategies (PRESS). Seven databases: MEDLINE (Ovid), PubMed (non-MEDLINE—Ovid), CINAHL (EBSCO), Embase (Ovid), Ageline, PsycINFO (Ovid), and Cochrane Central Register of Controlled Trials (CENTRAL) will be searched for studies that meet the inclusion criteria. Two reviewers will independently screen the results of the literature search using a two-step process. Eligible studies will undergo a quality assessment and data extraction. Disagreements will be resolved through consultation with a third reviewer. We will assess the quality of individual studies using the Mixed Methods Appraisal Tool (MMAT). The Grading of Recommendations, Assessment, Development and Evaluation (GRADE) will be used to summarize the strength of the quantitative evidence, and the Confidence in the Evidence from Reviews of Qualitative research (CERQual) tool to assess confidence in the qualitative syntheses.

**Discussion:**

This systematic review will summarize evidence-based knowledge for sensory practices, identify gaps in the literature, and inform an audit program for assessing the presence of sensory practices in the long-term care setting. The results will be relevant to policy makers, decision-makers, clinicians, and residents/families in long-term care settings.

**Systematic review registration:**

PROSPERO registration # CRD42017032330.

**Electronic supplementary material:**

The online version of this article (10.1186/s13643-018-0783-9) contains supplementary material, which is available to authorized users.

## Background

The five senses, including hearing, sight, taste, smell, and touch are reduced in older adults [1, 2]. Approximately 25–40% of individuals suffer from noticeable hearing loss after the age of 65, which increases to 80% after the age of 80 [1, 3, 4]. In addition, older adults may have increased difficulty distinguishing between certain colors and require more contrast between light and dark colors [1, 5]. A decline in taste buds typically occurs after the age of 40 in women and 50 in men [1, 6]. A diminishing sense of smell has more commonly been shown in adults over the age of 70 [7], which may lead to decreased interest in food and weight loss [1]. Impairment in smell also increases the safety risk for older adults since they may not be able to detect hazards (e.g., fires, gas leaks, etc.) [1]. Lastly, tactile changes such as decreased sensitivity to temperature and vibration may lead to increased injury (e.g., burns) due to slower reaction times to pain [1, 8].

Previous literature has commonly associated sensory decline with decreased quality of life and cognitive decline in older adults [9–18]. For instance, studies have shown that impairment in smell independently predicts all-cause 5-year mortality [10, 11] and taste deficits have been associated with nutritional compromise [17]. In addition, impairment in vision has been associated with depression [13, 14] whereas a slower gait speed has been correlated with hearing loss [15]. Moreover, tactile changes have been associated with a decreased nerve conduction velocity [18] and cognitive decline [19].

Furthermore, studies have investigated methods of modifying the physical environment to create a more enriching sensory environment for older adults living in long-term care settings. Such interventions included the following: adequate lighting [20], appropriate environmental temperatures [21], removal of unpleasant noises [22], presence of pleasant sounds (music) [23], and installation of multisensory environments including sensory gardens or Snoezelen rooms [24]. Other studies have focused on sensory interventions such as physical contact [25–28], animal therapy [29], aromatherapy and essential oils [30, 31], and nutrition [32–38].

A systematic review (SR) found several studies that focused on the physiological and psychological effects of health care environments [22]. These studies were conducted in a wide array of health care settings and patient populations (e.g., postoperative patients, patients waiting dental treatment, cardiac intensive care unit patients). There are also many studies that focus on the effect of long-term care environments for individuals with dementia [24, 39–42]. Despite the emerging research on the decline of the senses in older adults and the extensive research on quality of life, there is a paucity of literature that focuses on the relationship between sensory practices and the quality of life of residents in long-term care settings. Furthermore, no reviews to date have critically analyzed the impact of interventions related to the senses on the quality of life of older adults living in long-term care.

The objective of this review is to examine the relationship between the sensory practices and the quality of life of residents living in long-term care settings. To achieve this objective, we will conduct a mixed-methods SR in order to accommodate the different types of available studies in this area. These studies will include quantitative, qualitative, and mixed-methods designs. This review aims to address the following research question: What is the relationship between sensory practices and the quality of life of older residents living in long-term care settings?

## Methods

To develop this protocol, we used the Preferred Reporting Items for Systematic Review and Meta-Analysis Protocols (PRISMA-P) statement [43] (Additional file 1: PRISMA-P checklist). This protocol has been registered in the International Prospective Register of Systematic Reviews (PROSPERO). The study protocol registration number is: CRD42017032330.

We will use the Cochrane Handbook’s guidelines [44] and adhere to the Preferred Reporting Items for Systematic reviews and Meta-Analyses (PRISMA) standardized reporting format [45].

### Study eligibility criteria

We will evaluate qualitative, quantitative, and mixed-methods studies. The quantitative component of this review will explore the relationship between sensory practices and the quality of life of residents living in long-term care. The qualitative component will explore the views, perceptions, and beliefs of residents in relation to the impact of sensory practices on their own quality of life. To identify relevant studies, specific inclusion and exclusion criteria have been identified using the Population, Interventions, Comparators, Outcomes, and Study designs (PICOS): *Population*: This review will consider studies conducted with older adult residents living in long-term care settings. We will adapt the definition of “older person” depending upon the settings where the studies are conducted. For example, the World Health Organization’s definition for “older people” in Africa is 60 years of age or older [46]. There is a wide array of definitions for long-term care settings, with different living options and support needs for older adults [47]. The term “long-term care setting” is often used interchangeably with residential care facilities, assisted living, supportive housing, nursing homes, and/or long-term care homes. For the purposes of this systematic review, we will include all types of long-term care settings [47]. *Interventions*: This review will consider studies that evaluate sensory practices. These practices are defined as any practices implemented by a long-term care organization that focus on any of the five senses. Examples of such practices include but are not limited to auditory stimulation (used to enhance mood, promote relaxation, and cognition), fidget blankets (used to reduce the use of medication in agitation, particularly in older people with dementia), and modification of the physical layout of the environment (allowing residents to see and smell food as it is being prepared). *Comparators*: Studies will be eligible for inclusion whether or not they include comparison groups. *Outcomes*: For quantitative studies, our primary outcome is health-related quality of life (HRQOL) (e.g., Short-Form 36-item Health Survey). Secondary outcomes include resident and caregiver satisfaction/experience [48] (e.g., impact on mood, relaxation). For qualitative studies, we are looking for qualitative descriptions or themes related to the experiences of residents and/or their caregivers. *Study designs*: We will include a range of study designs including randomized and non-randomized studies, controlled before and after studies, historically controlled studies, retrospective or prospective cohort studies, mixed-methods studies, and qualitative studies (such as descriptive, ethnographic, narrative, case studies, and phenomenology).

### Data sources and search strategy

An expert health sciences librarian (LS) from the University of Ottawa worked with the research team to develop the search strategy. Table 1 outlines the MEDLINE (Ovid) search strategy. Prior to the execution of the search, a second health sciences librarian peer reviewed the search strategy using the Peer Review for Electronic Search Strategies (PRESS) [49]. Databases to be searched will include but are not limited to MEDLINE (Ovid), PubMed (non-MEDLINE—Ovid), CINAHL (EBSCO), Embase (Ovid), Ageline, PsycINFO (Ovid), and Cochrane Central Register of Controlled Trials (CENTRAL). Our preliminary database search retrieved a total of 4166 articles. Reference lists of included articles will also be hand-searched. The search will be undertaken by June 2018 and include all studies published until that date. We will also conduct a search of gray literature that relates to sensory practices. Sources of gray literature will include design manuals, policies and procedures, regulations, best practice standards, and guidelines of long-term care facilities. We will also look for resources available from government websites, long-term care facilities, products and services, and accreditation bodies. For other forms of gray literature, we will consider are new articles, dissertations, sensory guidebooks, and working papers.

**Table 1:** MEDLINE search strategy (as of July 2, 2017)

1 Assisted Living Facility/ (1179)	
2 (assist* adj3 living facilit*).tw. (610)	
3 exp. Residential Facilities/ (48568)	
4 exp. Homes for the Aged/ (12749)	
5 exp. Nursing Homes/ (36140)	
6 exp. Group Homes/ (934)	
7 (group adj2 home*).tw. (2176)	
8 (extended care adj2 facilit*).tw. (435)	
9 (long-term care adj2 facilit*).tw. (4889)	
10 (care adj2 (home* or facilit*)).tw. (45255)	
11 (rest adj2 home*).tw. (289)	
12 (residential adj2 (home* or care or facilit*)).tw. (5412)	
13 (geriatric adj2 (home* or unit* or facilit* or institution*)).tw. (2137)	
14 (nursing adj2 (home*1 or unit*1 or center*1 or centre*1)).tw. (30978)	
15 or/1-14 (102422)	
16 sensation*.tw. (37809)	
17 organoleptic*.tw. (2352)	
18 hearing.tw. (84736)	
19 audition.tw. (1378)	
20 smell.tw. (5995)	
21 (scent* or odo?r*).tw. (28630)	
22 olfaction.tw. (4470)	
23 taste*.tw. (25823)	
24 gustation*.tw. (323)	
25 touch.tw. (18195)	
26 taction*.tw. (13)	
27 (tactile adj2 sense*).tw. (200)	
28 Sensation/ (14880)	
29 sight.tw. (10205)	
30 Hearing/ or Vision, Ocular/ or Smell/ or Taste/ or Touch/ (85038)	
31 Sound/ or Noise/ or Lighting/ (41955)	
32 or/16-31 (301767)	
33 15 and 32 (1024)	

### Study screening and selection

Following the search, all identified citations will be collated and uploaded to Covidence (an online SR software application) [50] where duplicates will be deleted. We will use a two-step process to screen the results of the literature search as follows: (1) title and abstract screening for inclusion (level 1) and (2) full-text screening for inclusion (level 2). Level 1 and level 2 screening will be performed independently by two reviewers (MC, DC). A third reviewer (CB) will be consulted in the case of inclusion and exclusion conflicts. Full-text studies that do not meet the inclusion criteria will be excluded and reasons for exclusion will be documented. For non-English articles, we will attempt to find an English translation. For French articles, CB will conduct the abstract and full-text screening to determine inclusion eligibility. Search results will be summarized in a PRISMA study flow diagram [45].

### Risk of bias and quality assessment

All included (quantitative, qualitative, and mixed methods) studies will be critically appraised for methodological quality using the Mixed Methods Appraisal Tool (MMAT) [51, 52]. The MMAT provides comprehensive guidelines and simplicity for summarizing overall quality across a range of study designs (e.g., quantitative, qualitative or mixed methods). Each study will be assigned an overall quality score, using asterisks representing the quality appraisal of each study. For qualitative and quantitative studies, there are four specific criteria and the scores will vary from 25% (*) when one criterion is met to 100% (****) when all four criteria are met [51]. For mixed-methods studies, there are three criteria, and the scores vary from 50% (**) when one criterion is met to 100% (****) when all three criteria are met [51]. Concomitant appraisal of qualitative and quantitative aspects of each mixed-methods study is required in the MMAT tool, with the overall score not exceeding the lowest component score [51]. Two reviewers (MC, DC) will independently assess the quality of studies, and disagreement will be resolved through consensus, and discussion with a third reviewer (CB). We will construct a risk of bias table to present the results. We will also conduct a sensitivity analysis where the studies perceived to be of lower quality (lower MMAT scores) will be compared with higher quality (higher MMAT score) [51]. Studies will not be excluded on quality grounds, but lower quality studies will be reviewed to see if they alter the outcome of the synthesis.

### Data extraction

Two reviewers (DC, MS) will independently extract the data from each included study using a standardized data abstraction form. The data extraction form will be piloted on the first ten studies and necessary revisions will be made accordingly. Data from each included study will include the following: study characteristics (year of publication, authors, country of study), study objectives, study design, target population, sample size, description of the practice, outcome measures (if available or applicable), and study results. The data extraction tool will be adapted as needed, based on the nature of the included studies. Any disagreements that arise between the reviewers will be resolved through discussion or with a third reviewer (CB). Authors of the studies will be contacted to request missing or additional data where required and will be given 30 days to respond.

### Data synthesis

The quantitative and the qualitative extracted data will be analyzed separately using different synthesis methods. The data extracted from the eligible studies will be grouped and analyzed by study design (e.g., quantitative, qualitative, mixed methods). Data will be aggregated and analyzed according to each of the senses (e.g., hearing, sight, taste, touch, smell). Studies that include more than one sense will be aggregated and analyzed separately.

#### Stage 1 data synthesis of the quantitative evidence

Statistical analysis will be performed using RevMan5 [53]. Appropriate measures of treatment effect will be determined based on the type of data collected in the included studies. We will analyze categorical data using odds ratio (OR) and risk ratio (RR). Continuous data will be analyzed from means and standard deviations wherever possible. When means and standard deviations are not reported, we will use other available data (for example, confidence intervals, *t* values, *P* values) and appropriate methods described in the Cochrane Handbook for Systematic Reviews of Interventions [44] to calculate the means and standard deviations. Heterogeneity will be assessed statistically using the standard chi-squared and *I*-squared tests. Subgroup analyses (e.g., residents with dementia) will be conducted where there is sufficient data to investigate.

#### Stage 2 data synthesis of the qualitative/mixed-methods evidence

We will conduct a thematic synthesis informed by the “framework” analysis approach to analyze and synthesize the data [54]. The five stages of analysis include the following: (1) becoming familiar with the content of the data, (2) identifying a thematic framework (key issues, concepts and themes), (3) systematically applying the thematic framework to the data, (4) rearranging the data according to the appropriate thematic reference, and (5) identifying the key characteristics of the data. The text of each study will be indexed, by at least two reviewers (MC, DC), using the codes relating to the themes and sub-themes of the thematic framework. The emerging themes and concepts will be transferred to analysis tables, so that the columns and rows of the table reflect the studies and sub-themes. The table will enable the team to compare the results obtained in different studies across different themes and sub-themes and to compare the findings of different studies for each theme. A qualitative review across the studies will allow us to not only examine what designs are successful but also evaluate what it is about different practices that may work and under what circumstances [55].

Both syntheses performed will not be combined but they will be presented separately. The results of the syntheses will be interpreted and the recommendations will take account of all the quantitative and qualitative findings.

### Assessing overall confidence in the evidence

We will use the Grading of Recommendations, Assessment, Development and Evaluation (GRADE) approach to assess the confidence in the evidence of the quantitative syntheses [56–58]. We will present our GRADE assessments in a summary of findings table. The summary of findings table will present the following information where appropriate: ranking of the quality of the evidence based on study limitations (risk of bias), indirectness, inconsistency, imprecision, and publication bias.

We will use the Confidence in the Evidence from Reviews of Qualitative research (CERQual) tool to assess confidence in the qualitative syntheses [59]. Specifically, we will assess four components: (1) methodological limitations of included studies, (2) relevance of contributing studies to the research question, (3) coherence of study findings, and (4) adequacy of the data supporting the study findings. Two reviewers (MC, DC) will independently assess each component. We will rate overall assessment of confidence as high, moderate, low, or very low and provide rationale for the rating. We will assign high confidence if it is “highly likely,” moderate confidence if it is “likely,” low confidence if it is “possible,” and very low confidence if it is “not clear” that the review finding is a reasonable representation of the topic [59]. We will present our CERQual findings in a CERQual Qualitative Evidence Profile [59].

## Discussion

Understanding sensory practices in long-term care settings remains a relatively new research topic, and there is a paucity of literature that investigates all five senses. This SR will summarize the sensory practices used in organizations, determine the gaps in the existing literature, disseminate research findings, and make recommendations for future research priorities. We will also use our SR findings (i.e., tabulated data, quality of evidence, and confidence in the evidence) to inform the content development of an audit tool for long-term care organizations to use in assessing their sensory environment. These results will be relevant for policy makers, decision-makers, clinicians, and residents/families in long-term care settings.

## Additional file

Additional file 1: ᅟPRISMA-P checklist (DOCX 40 kb)

## Abbreviations

CERQual: Confidence in the Evidence from Reviews of Qualitative research
GRADE: Grading of Recommendations, Assessment, Development and Evaluation
MMAT: Mixed Methods Appraisal Tool
PICOS: Population, Interventions, Comparators, Outcomes, and Study designs
PRESS: Peer Review for Electronic Search Strategies
PRISMA: Preferred Reporting Items for Systematic reviews and Meta-Analyses
PRISMA-P: Preferred Reporting Items for Systematic Review and Meta-analysis Protocols
PROSPERO: International Prospective Register of Systematic Reviews
SR: Systematic review
